# A novel *PAX3* mutation in a Japanese boy with Waardenburg syndrome type 1

**DOI:** 10.1038/hgv.2016.5

**Published:** 2016-03-03

**Authors:** Yu Yoshida, Rieko Doi, Kaori Adachi, Eiji Nanba, Isamu Kodani, Kazuo Ryoke

**Affiliations:** 1 Division of Oral and Maxillofacial Biopathological Surgery, Department of Medicine of Sensory and Motor Organs, School of Medicine, Tottori University Faculty of Medicine, Yonago, Japan; 2 Division of Functional Genomics, Research Center for Bioscience and Technolog y, Tottori University, Yonago, Japan

## Abstract

Waardenburg syndrome type 1 (WS1) is a rare autosomal dominant disorder characterized by hair hypopigmentation, abnormal iris pigmentation, and congenital hearing loss. WS1 is caused by mutations in paired box gene 3 (*PAX3*). We identified a novel *PAX3* mutation (c.1107 C>G, p.Ser369Arg) in a Japanese WS1 patient showing abnormal right iris pigmentation, right-sided congenital hearing loss, synophrys, incomplete left cleft lip, and cryptorchidism.

Mutations in paired box gene 3 (*PAX3*, NM_181457.3.) are responsible for Waardenburg syndrome type 1 (WS1; OMIM 193500),^1,2^ which is clinically characterized by hair hypopigmentation, abnormal iris pigmentation, and congenital hearing loss. Its prevalence is estimated to be 1/42,000. Furthermore, it is responsible for 1–3% of total congenital deafness.^3^ Diagnosis of WS1 is established by clinical findings in most individuals. Five major white forelock and hair hypopigmentation are one pair, dsytopia canthorum and W index>1.95 are one pair. (• congenital hearing loss • white forelock, hair hypopigmentation • abnormal iris pigmentation • dystopia canthorum, W index>0.95 • affected first-degree relative) and minor diagnostic criteria for WS have been proposed by the Waardenburg Consortium.^4^ An individual must have two major criteria or one major plus two minor criteria to be diagnosed with WS.^4,5^ *PAX3* is one of the nine human PAX genes encoding DNA-binding transcription factors.^6^ *PAX3* has 10 exons and encodes a paired box by exons 2–4 and a homeobox by exons 5 and 6.^6^

The patient was a 10-month-old Japanese boy born at 33 weeks of gestation with a (very low) birth weight of 1,250 g. He was referred to our hospital because of an incomplete left cleft lip. Body weight at the time of the first visit was 6,700 g at 10 months of age. Although his physique was petite, his nutritional status was good. The patient showed pigmentation abnormalities of the right iris, ocular hypertelorism (W index: 2.00), right-sided congenital hearing loss, synophrys, incomplete left cleft lip, and cryptorchidism. He was diagnosed with WS1 on the basis of clinical findings and the criteria proposed by the Waardenburg Consortium.^4^ According to the parents, his family members, including the proband and mother, showed clinical features consistent with WS1 (Figure 1). The proband is indicated by an arrow (Figure 1a:21). His mother’s hair was speckled with grey. However, she showed no other symptoms (Figure 1a:20). His mother’s aunt showed a lateral iris pigmentation abnormality (Figure 1a:14), and his mother’s elder sister showed a lateral iris pigmentation abnormality and bilateral congenital hearing loss (Figure 1a:19).

Surgery to repair the cleft lip was performed at 14 months, at a weight of 6,900 g. The preoperative assessment was not problematic.

His parents wanted the patient to undergo a genetic test for *PAX3*. For the genetic diagnosis, blood samples were obtained from the patient after informed consent was obtained from the parents. Genomic DNA was extracted from the blood samples using a standard protocol. All 10 exons and exon–intron junctions of the *PAX3* gene were amplified from genomic DNA by PCR using primers designed with primer software (Genetyx software, Genetyx, Shibuya, Japan). The primers were designed to include an intron of 50 bp surrounding the exon. The sequencing was performed using forward and reverse primers. All the primer sequences are available on request. Sequencing was performed with a BigDye Terminator v3.1 Cycle Sequencing Kit and a 3500×l Genetic Analyzer capillary sequencer (Life Technologies, Carlsbad, CA, USA). Mutation analysis revealed a novel missense mutation (c.1107 C>G, p.Ser369Arg) in exon 7 (Figure 2a).

The mutation was predicted to be pathogenic using PolyPhen-2 (probably damaging, Hum Var 0.958, Hum Div 0.776).^7^ MutationTaster indicated that the mutation was a disease-causing mutation (model: simple_aae, prob: 0.999980746176575).^8^ Parental analysis was refused. The mutated Ser369 residue is evolutionarily conserved from fish to humans (Figure 2b). Ser369 resides in a highly conserved transactivation domain that interacts with DNA (Figure 2c). These symptoms are believed to arise due to the mutation, which prevents the formation of a covalent disulfide bond (S–S bond), resulting in protein instability.

The variant p.Ser369Arg is located in exon 7. To date, other WS-related mutations have been reported in this exon.^9–12^ Most of the reported mutations in *PAX3* are localized in exons 2–6. Deletions comprise two whole gene deletions and two intragenic deletions of exon 7, and exons 8 and 9.^12,13^ There is no correlation between the mutation type (missense, nonsense, or deletion) and severity of the phenotype.^13^ A few mutations of PAX3 have been analyzed for their functional consequences.^1,2,13^ In addition, functional studies are required to assess the exact molecular mechanisms induced by the c.1107C>G mutation. Cleft lip and palate are low-penetrance features of WS1 and have been found in 2.8% of the reported cases.^14^

In conclusion, we identified a novel *PAX3* mutation (c.1107 C>G, p.Ser369Arg) in a Japanese boy showing pigmentation abnormality in the right iris, right-sided congenital hearing loss, synophrys, incomplete left cleft lip, and cryptorchidism due to WS1. This finding further broadens the existing knowledge of mutations of the *PAX3* gene in WS1 patients, enabling clinicians and genetic counsellors to offer better clinical services. WS1 pathogenesis should be taken into account when treating WS1 infants with a cleft lip.

## Figures and Tables

**Figure 1:**
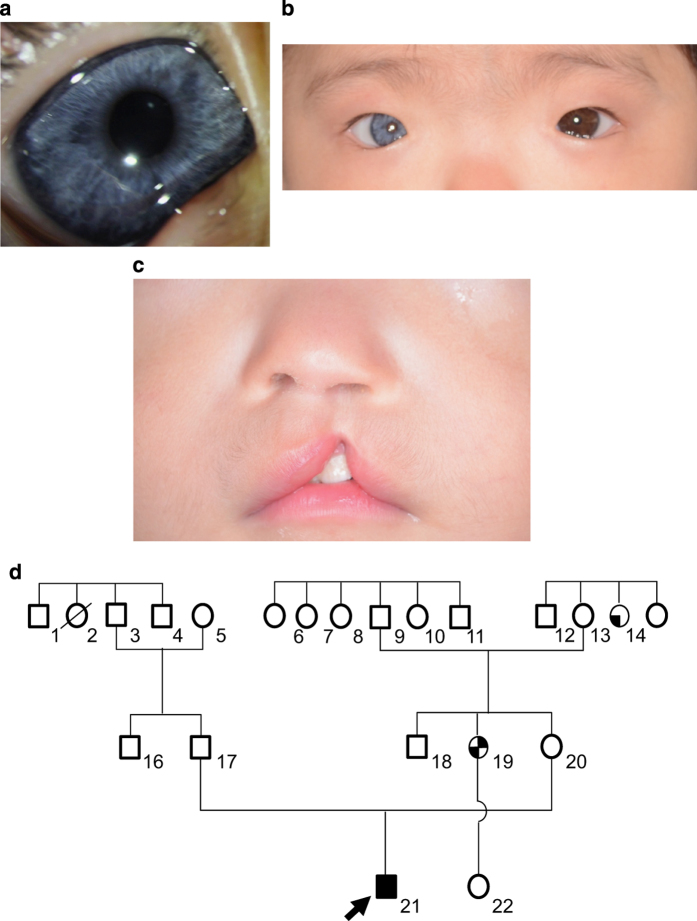
(**a**) Family pedigree of the WS1 patient. Squares indicate males and circles indicate females. Shading in these symbols corresponds to symptoms as follows: upper left quadrant, ocular hypertelorism; lower left, abnormal iris pigmentation; upper right, hearing loss; and lower right, synophrys. (**b**) Abnormal pigmentation of the right iris of the patient. (**c**) Ocular hypertelorism (W index: 2.00) of the patient. (**d**) Incomplete left cleft lip of the patient.

**Figure 2:**
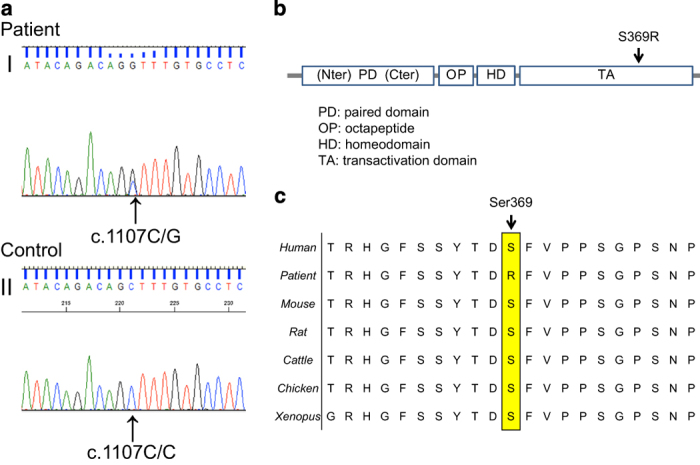
(**a**) Sequence chromatograms of the WS1 patient and a control. I: A heterozygous change, c.1107C>G, was identified in the affected boy. II: Healthy controls are wild-type at this position. (**b**) Schematic diagram of the *PAX3* gene. The arrow indicates the mutated gene. The reported missense mutation identified in the WS1 patient is presented. (**c**) Cross-species multiple alignment of *PAX3* protein sequences, showing evolutionary conservation of the altered amino acid, Ser369.

## References

[bib1] Baldwin CT , Hoth CF , Amos JA , da-Silva EO , Milunsky A . An exonic mutation in the HuP2 paired domain gene causes Waardenburg’s syndrome. Nature 1992; 355: 637–638. 1347149 10.1038/355637a0

[bib2] Hoth CF , Milunsky A , Lipsky N , Sheffer R , Clarren SK , Baldwin CT . Mutation in the paired domain of the human *PAX3* gene cause Klein-Waardenburg syndrome(WS-III) as well as Waardenburg syndrome Type-I(WS-I). Am J Hum Genet 1993; 52: 455–462. 8447316 PMC1682157

[bib3] Read AP , Newton VE . Waardenburg syndrome. J Med Genet 1997; 34: 656–665. 9279758 10.1136/jmg.34.8.656 PMC1051028

[bib4] Farrer LA , Grundfast KM , Amos J , Arnos KS , Asher JH Jr , Beighton P et al. Waardenburg Syndrome (WS) type I is caused by defects at multiple loci, one of which is near ALPP on chromosome 2: First report of the WS consortium. Am J Hum Genet 1992; 50: 902–913. 1349198 PMC1682585

[bib5] Dourmishev AL , Dourmishev LA , Schwartz RA , Janniger CK . Waardenburg syndrome. Int J Dermatol 1999; 38: 656–663. 10517681 10.1046/j.1365-4362.1999.00750.x

[bib6] Birrane G , Soni A , Ladias JAA . Structual basis for DNA recognition by the human *PAX3* homeodomain. Biochemistry 2009; 48: 1148–1155. 19199574 10.1021/bi802052y

[bib7] Adzhubei IA , Schmidt S , Peshkin L , Ramensky VE , Gerasimova A , Bork P et al. A method and server for predicting damaging missense mutations. Nat Methods 2010; 7: 248–249. 20354512 10.1038/nmeth0410-248 PMC2855889

[bib8] Schwarz JM , Cooper DN , Schuelke M , Seelow D . MutationTaster2: mutation prediction for the deep-sequencing age. Nat Methods 2014; 11: 361–362. 24681721 10.1038/nmeth.2890

[bib9] Baldwin CT , Hoth CF , Macina RA , Milunsky A . Mutations in PAX3 that cause Waardenburg syndrome type I: Ten new mutations and review of the literature. Am J Med Genet A 1995; 58: 115–122. 10.1002/ajmg.1320580205 8533800

[bib10] Carey ML , Friedman TB , Asher JH Jr , Innis JW . Septo-optic dysplasia and WS1 in the proband of a WS1 family segregating for a novel mutation in PAX3 exon 7. J Med Genet 1998; 35: 248–250. 9541113 10.1136/jmg.35.3.248 PMC1051252

[bib11] Pingault V , Ente D , Dastot-Le Moal F , Goossens M , Marlin S , Bondurand N et al. Review and update of mutations causing Waardenburg syndrome. Human Mutation 2010; 31: 391–406. 20127975 10.1002/humu.21211

[bib12] Jalilian N , Tabatabaiefar MA , Farhadi M , Bahrami T , Noori-Daloii MR . A novel mutation in the *PAX3* gene causes Waardenburg syndrome type I in an Iranian family. Int J Pediatr Otorhinolaryngol 2015; 79: 1736–1740. 26279250 10.1016/j.ijporl.2015.07.039

[bib13] Wildhardt G , Zirn B , Graul-Neumann LM , Wechtenbruch J , Suckfüll M , Buske A et al. Spectrum of novel mutations found in Waardenburg syndrome type1 and 2: Implications for molecular genetic diagnosis. BMJ Open 2013; 3: e001917. 10.1136/bmjopen-2012-001917 PMC3612789 23512835

[bib14] Tamayo ML , Gelvez N , Rodriguez M , Florez S , Varon C , Medina D et al. Screening program for Waardenburg syndrome in Colombia: Clinical definition and phenotypic variability. Am J Med Genet A 2008; 146A: 1026–1031. 18241065 10.1002/ajmg.a.32189

